# Recent Advances in the Synthesis of Hydrogenated Azocine-Containing­ Molecules

**DOI:** 10.1055/s-0036-1589500

**Published:** 2017-08-01

**Authors:** Anna V. Listratova, Leonid G. Voskressensky

**Affiliations:** RUDN UniversityMiklukho-Maklaya St. 6, Moscow, 117198Russian Federationvoskresenskiy_lg@rudn.university.ru

**Keywords:** azocine, ring-closing metathesis, cycloaddition, ring expansion, Heck reaction, domino reaction

## Abstract

This review covers recent advances in synthesis of azocine-containing systems. The most approaches towards azocines are discussed.

1 Introduction

2 Ring-Expansion Reaction

2.1 Ring-Expansion Reaction of Cyclopentane Containing the 1,4-Diketone Moiety with Primary Amines (from 5 to 8)

2.2 Ring-Expansion Reaction of Annulated Tetrahydropyridines under the Action of Activated Alkynes (from 6 to 8)

2.3 Reductive Ring-Expansion Reaction of Cyclic Oximes

2.4 Other Ring-Expansion Reactions

3 Heck Reaction

4 Cycloaddition

5 Ring-Closing Metathesis (RCM)

6 Cyclization

6.1 Metal-Catalyzed Cyclization

6.2 Radical Cyclization

6.3 Friedel–Crafts Cyclization

6.4 Other Examples of Cyclizations

7 Microwave- and Photo-Assisted Reactions

8 Other Methods

8.1 Cascade and Tandem Reactions

8.2 Aldol Condensation

8.3 Thermolysis

8.4 Ring Opening

8.5 Other Methods

9 Conclusion

## 1 Introduction

The chemistry of annulated azocines has not been explored in detail owing to the lack of efficient methods for their synthesis. The only exception is azocinoindoles, which have been investigated extensively due to the great number of alkaloids with an azocinoindole fragment in their structure. This review highlights most recent approaches towards annulated azocine derivatives published after the year 2009; the previous review was published in 2008. 1


**Scheme 1** Synthesis of benzo[ *c* ]azocines


## 2 Ring-Expansion Reactions

### 2.1 Ring-Expansion Reaction of Cyclopentane Containing the 1,4-Diketone Moiety with Primary Amines (from 5 to 8)


**Scheme 2** Synthesis of a pyrido[2,3- *c* ]azocine



**Scheme 3** Synthesis of a pyrido[3,4- *c* ]azocine


In 2006 the Cristoffers group 2 discovered a novel bismuth-catalyzed ring-expansion reaction of 1,4-diketones with primary amines that furnished an eight-membered ring. In 2011, they extended their relatively simple method to the synthesis of annulated azocines. Thus, starting from ethyl 1-oxo-indane-2-carboxylate **1** , containing a 1,4-diketone motif, and primary amines under the bismuth-catalyzed ring-expansion reaction conditions gave benzo[ *c* ]azocine derivatives **2** in moderate yields (Scheme 1 ). 3 It was also shown that, in some cases, the presence of bismuth nitrate was not essential. 3

A bismuth-free strategy of ring enlargement was also successful in the case of regioisomeric pyrido[ *c* ]azocines. 4 Synthesized from commercially available materials, three cyclopentapyridine derivatives **3** , **6** , and **9** , containing β-oxo ester moieties, were alkylated with phenacyl bromide to give 1,4-diketones **4** , **7** , and **10** . The latter were subjected to ring-expansion reactions with methylamine giving pyrido[2,3- *c* ]azocine **5** (Scheme 2 ), pyrido[3,4- *c* ]azocine **8** (Scheme 3 ), and pyrido[3,2- *c* ]azocine **11** (Scheme 4 ) in 36–64% yield.


**Scheme 4** Synthesis of a pyrido[3,2- *c* ]azocine


In 2015, a six-step sequence for the synthesis of regioisomeric thieno[ *c* ]azocines started from commercially available bromothiophenecarboxylic acids was worked out. 5 Isopropyl esters of the bromothiophenecarboxylic acids were subjected to Heck reaction followed by catalytic hydrogenation and Dieckmann condensation giving the cyclic β-oxo esters **12** , **15** , and **18** , alkylation of which with phenacyl bromide led to 1,4-diketones **13** , **16** , and **19** . The following step, a bismuth-catalyzed ring expansion of cyclopentathiophene derivatives **13** , **16** , and **19** with methylamine, produced the target tetrahydrothieno[3,2- *c* ]azocine **14** (Scheme 5 ), tetrahydrothieno[3,4- *c* ]azocine **17** (Scheme 6 ), and tetrahydrothieno[2,3- *c* ]azocine **20** (Scheme 7 ). Overall yields for the final products were 25%, 16%, and 12%, respectively.


**Scheme 5** Synthesis of a tetrahydrothieno[3,2- *c* ]azocine



**Scheme 6** Synthesis of a tetrahydrothieno[3,4- *c* ]azocine



**Scheme 7** Synthesis of a tetrahydrothieno[2,3- *c* ]azocine


### 2.2 Ring-Expansion Reaction of Annulated Tetrahydropyridines under the Action of Activated Alkynes (from 6 to 8)

In 2002, an alkyne-induced ring-expansion reaction of annulated tetrahydropyridines leading to the formation of azocine rings was found. 6 It is presumed that ring-expansion reaction involves the Michael addition of the tertiary N-atom in the (hetero)annulated pyridine system to the triple bond of the activated alkyne, followed by a nucleophilic substitution (S _N_ ) reaction in zwitterionic intermediate **A** (Scheme 8 ).

Over the last 8 years this method was successfully applied to the synthesis of various annulated azocines: triazolopyrimido[4,5- *d* ]azocines **21** , 7 8 tetrahydro[1]benzothieno[3,2- *d* ]azocines **22** , 9 hexahydropyrimido[4,5- *d* ]azocines **23** and -[5,4- *d* ]azocines **24** , 10 tetrahydropyrimido[4,5- *d* ]azo­cines **25** , 11 tetrahydrobenzofuro[3,2- *d* ]azocine **26** , 12 tetrahydrothieno[2,3- *d* ]azocines **27** , 13 tetrahydroazocino[5,4- *b* ]indoles **28** , 14 15 hexahydropyrimidothieno[3,2- *d* ]azocines **29** , 16 17 and benzo[ *d* ]azocines **30** , 18 including systems obtained for the first time, tetrahydrothieno[3,2- *d* ]azocines **31** 19 and tetrahydrochromeno[4,3- *d* ]azocine **32** (Figure 1 ). 20


**Scheme 8** Plausible mechanism for the transformation of the tetrahydropyridine ring



**Figure 1** Series of annulated azocines obtained by alkyne-induced ring-expansion reaction


Attempts to combine the aforesaid ring expansion and Ugi or Ugi-azide transformations into a single multicomponent reaction succeeded and provided thieno[3,2- *d* ]azocine **33** 21 (Scheme 9 ) and tetrazolyl-substituted benzo[ *d* ]azocine **34** 18 (Scheme 10 ) in moderate yields.


**Scheme 9** Combination of ring-expansion and Ugi reactions



**Scheme 10** Combination of ring-expansion and Ugi-azide reactions


### 2.3 Reductive Ring-Expansion Reaction of Cyclic Oximes


**Scheme 11** Synthesis of azocine systems via reductive ring-expansion reaction of cyclic oximes


Using a method devised by Cho, Tokuyama, and co-workers, regiocontrolled reductive ring-expansion of cyclic oxime with diisobutylaluminum hydride gave benzo[ *b* ]azocine **35** and dibenzo[ *b* , *f* ]azocine **36** in high yields as a single regioisomer with the nitrogen atom located in the position neighboring the aromatic ring (Scheme 11 ). 22 23 24

Based on this ring-expansion reaction of oximes, a concise synthesis of 17β-HSD3 inhibitor with a dibenzoazocine skeleton was carried out. 25 All attempts to convert oximes **37a** , **b** into a single regioisomer failed, hence a mixture of dibenzoazocines **38a** and **39a** in the ratio 2:1 or dibenzoazo­cines **38b** and **39b** in the ratio 6:1 was used. After acylation of dibenzoazocines **38** and **39** the obtained regioisomers **40** and **41** were separated. Compound **40a** was coupled under the Suzuki–Miyaura coupling conditions to provide 17β-HSD3 inhibitor **42** in 70% yield. Desulfurization of **42** with Raney Ni gave also 17β-HSD3 inhibitor **43** (Scheme 12 ). Since 17β-hydroxysteroid dehydrogenase type 3 (17βHSD3) is an enzyme involved in testosterone biosynthesis, inhibitors of 17β-HSD3 could provide new medicines for the treatment of prostate cancer.


**Scheme 12** A concise synthesis of 17β-HSD3 inhibitor


### 2.4 Other Ring-Expansion Reactions

Treatment of 1-vinyl-substituted indolinium salt **44** in refluxing THF with the Hoveyda–Grubbs second-generation catalyst (H-G II) resulted in allylic rearrangement to give azocino[5,4- *b* ]indole **45** , the product of ring expansion, in 44% yield (Scheme 13 ). 26

A conceptually new and elegant strategy for the construction of 1 *H* -azocino[5,4- *b* ]indoles **47** and **48** via a gold-catalyzed ring expansion of 2-propargyl-β-tetrahydrocarbolines **46** was developed by Zhang and co-workers. 27 The azocinoindoles **47** and **48** were obtained in moderate to excellent yields. The method features mild conditions and wide functional group tolerance (Scheme 14 ).


**Scheme 13** Allyl rearrangement of 1-vinyl-substituted indolinium salt



**Scheme 14** Synthesis of 1 *H* -azocino[5,4- *b* ]indoles via a gold-catalyzed ring expansion of 2-propargyl-β-tetrahydrocarbolines; Au(I) = [AuNTf _2_ (PPh _3_ )]


Binaphthyl-azocines **50** were synthesized by the direct copper-catalyzed ring-expansion reaction of binaphthyl-azepines **49** and α-diazocarbonyl reagents. 28 This transformation is considered to be an example of a [1,2-]Stevens rearrangement and presents a facile access to binaphthyl-azo­cines in moderate to high yields (Scheme 15 ).


**Scheme 15** Synthesis of binaphthyl-azocines


Naphtho[1,8- *ef* ]pyrimido[4,5- *b* ]azocines **53** were prepared from acenaphthoquinone ( **51** ) and 6-aminouracil derivatives **52** in high yields. 29 The ring expansion was carried out as a one-pot process and included two steps: the addition reaction of starting compounds and the subsequent oxidation cleavage of the intermediate in the presence of Pd(OAc) _4_ (Scheme 16 ).


**Scheme 16** Synthesis of naphtho[1,8- *ef* ]pyrimido[4,5- *b* ]azocines


Azocine **55** and annulated azocine **57** were produced by a ring-expansion transformation of the piperidine ring of compounds **54** and **56** under the action of methyl propyn­oate and dimethyl butynedioate, respectively (Scheme 17 ). 30


**Scheme 17** Synthesis of azocines via transformation of the piperidine ring


## 3 Heck Reaction

The intramolecular Heck reaction is considered to be one of the most useful methods for the construction of medium-sized heterocycles due to its functional group tolerance and high stereoselectivity. In 2009, the Majumdar group developed an interesting and simple procedure for the synthesis of various annulated azocines from unactivated allylic substrates using a combination of two reactions, the aza-Claisen rearrangement and a palladium-catalyzed intramolecular Heck reaction. Thus, an appropriate substrate **59** , prepared from *N* -allylanilines **58** by aza-Claisen rearrangement, tosylation, and alkylation with 2-bromobenzyl bromides, was subjected to the intramolecular Heck reaction to give exo-Heck cyclized products, dibenzo[ *b* , *f* ]azo­cines **60** , in 72–79% yields (Scheme 18 ). 31


**Scheme 18** Synthesis of dibenzo[ *b* , *f* ]azocines via the intramolecular Heck reaction


This strategy was successfully used for the synthesis of dibenzo[ *b* , *f* ]azocinones. 32 The precursors **61** for the Heck reaction were obtained from *N* -allyl-substituted anilines by aza-Claisen rearrangement, tosylation, and amidation with 2-iodobenzoyl chloride. The products of the Heck reaction depended on the conditions used. It was shown that Jeffrey­’s two-phase protocol 32 led to endocyclic product **63** whereas phosphine-assisted standard conditions yielded exocyclic products **62** (Scheme 19 ).


**Scheme 19** Synthesis of dibenzo[ *b* , *f* ]azocines via the intramolecular Heck reaction


The same combined aza-Claisen rearrangement and intramolecular Heck reaction was successfully applied to the synthesis of coumarin- or quinolone-annulated azocines **65** 33 and pyrimidoazocines **67** . 34 The precursors **64** for the Heck reaction were synthesized using aza-Claisen rearrangement of *N* -allylcoumarins or *N* -allylquinolones followed by alkylation with benzyl bromides. The intramolecular Heck reaction afforded azocine **65** in 75–79% yields (Scheme 20 ).


**Scheme 20** Synthesis of coumarin- and quinolone-annulated azocines


In the case of pyrimido[5,4- *b* ]azocines **67** , the substrates **66** for the Heck reaction were prepared from 1,3-dialkyl-5-bromouracils by reaction with allylamine, subsequent aza-Claisen rearrangement, tosylation, and alkylation with 2-bromobenzyl bromide. Pyrimidoazocines **67** were obtained in high yields (Scheme 21 ).


**Scheme 21** Synthesis of pyrimido[5,4- *b* ]azocines



**Scheme 22** Construction of dibenzoazocinones and coumarin- and quinolone-annulated azocinones via Pd-mediated reductive Mizoroki–Heck reaction


The Majumdar group achieved another efficient and straightforward method for the construction of dibenzoazo­cinones **72** and coumarin- and quinolone-annulated azocinones **73** via Pd-mediated reductive Mizoroki–Heck reaction. 35 The starting *N* -methyl or *N* -ethyl *o* -substituted amines **68** and **71** reacted with 2-iodophenylacetyl chloride giving amides **69** and **72** , which were subjected to Heck reaction producing the 8-exocyclized products **70** and **73** in 42–60% yields (Scheme 22 ).


**Scheme 23** Synthesis of tetracyclic azocine derivatives



**Scheme 24** Synthesis of benzo[4,5]azocino[3,2,1- *hi* ]indoles



**Scheme 25** Synthesis of benzo[ *e* ]imidazo[4,5,1- *kl* ][1]benzoazocines


Kim and co-workers obtained tetracyclic azocine systems **77** from indole derivatives **76** by applying the intramolecular palladium-catalyzed Heck reaction. 36 The required starting materials were synthesized by the reaction of Baylis–Hillman acetates **74** and indoles **75** (Scheme 23 ).


**Scheme 26** Synthesis of benzo[4,5]azocino[1,2,3- *jk* ]carbazole



**Scheme 27** Synthesis of azocino[3,4,5- *cd* ]indoles by Pd-catalyzed microwave-assisted Heck reaction


Application of this protocol to compounds **79** , **82** , and **85** , derived from the reaction of Baylis–Hillman acetate **74** with isatins **78** , benzimidazoles **81** , and carbazole **84** , respectively, resulted in the formation of tetra(penta)cyclic azocines, benzo[4,5]azocino[3,2,1- *hi* ]indoles **80** (Scheme 24 ), benzo[ *e* ]imidazo[4,5,1- *kl* ][1]benzoazocines **83** (Scheme 25 ), and benzo[4,5]azocino[1,2,3- *jk* ]carbazole **86** (Scheme 26 ). 37

Martin and co-workers synthesized azocino[3,4,5- *cd* ]indoles **88** + **89** and **91** by Pd-catalyzed microwave-assisted Heck reaction from allylamine derivative **87** or enamine **90** (Scheme 27 ). 38

The unusual Heck product azocino[4,3- *b* ]indole **93** , resulting from an apparent 7- *endo* -cyclization with inversion of the ethylidene configuration, was obtained from cyclohepta[ *b* ]indole **92** in 43% yield (Scheme 28 ). 39

A readily separable mixture (1.3:1.0) of bridged benzoazocines **95** and **96** was formed through an intramolecular microwave-assisted Heck cyclization from azepine derivative **94** (Scheme 29 ). 40

Dibenzo[ *b* , *f* ]azocine **98** was produced via microwave-assisted Heck coupling from bifunctional precursor **97** prepared by vinylation of bromobenzaldehyde and subsequent reductive imine condensation with the relevant 2-bromoaniline. 41 Dibenzo[ *b* , *f* ]azocine **98** was also synthesized by a Suzuki–Heck cascade and also by a one-pot preparation (Scheme 30 ). 41


**Scheme 28** Synthesis of an azocino[4,3- *b* ]indole



**Scheme 29** Synthesis of bridged azocines through an intramolecular microwave-assisted Heck reaction



**Scheme 30** Synthesis of dibenzo[ *b* , *f* ]azocine by a Suzuki–Heck cascade


Starting from propargylamide **99** , a novel protocol for the tandem Heck–Suzuki reaction was used for the construction of the benzoazocines **100** and **101** (Scheme 31 ). 42


**Scheme 31** Synthesis of benzoazocines **100** and **101** via a Heck–Suzuki cascade


## 4 Cycloaddition

In 2009, Rovis and co-workers developed the first enantioselective rhodium-catalyzed [4+2+2] cycloaddition of terminal alkynes **102** and dienyl isocyanates **103** leading to the formation of bicyclic azocines **104** and **105** . 43 Pyrrolo[1,2- *a* ]azocines **104** and **105** were obtained in good to high yields and excellent enantioselectivity (Scheme 32 ). The geometry of the diene moiety had a significant effect on the selectivity of the products and used pure ( *E* )-diene was used as the starting substrate.


**Scheme 32** Synthesis of pyrrolo[1,2- *a* ]azocines


A new and simple synthesis for azocine derivatives **108** by [6+2] cycloaddition reaction was suggested by Saito and co-workers. 44 Electron-deficient allenes **107** reacted with 2-vinylazetidine **106** giving azocines **108** in moderated yields (Scheme 33 ).


**Scheme 33** Synthesis of azocine derivatives


Louie and co-workers demonstrated in 2012 that azetidin-3-ones **110** under the action of diynes **109** underwent a Ni/IPr-catalyzed cycloaddition reaction leading to dihydroazocinones **111** (Scheme 34 ). 45 The method involves a Csp ^2^ –Csp ^3^ bond-cleavage step that proceeds at low temperatures.


**Scheme 34** Synthesis of dihydroazocinone derivatives


In 2013, Louie and co-workers reported the Ni/P( *p* -Tol) _3_ -catalyzed cycloaddition of 1,3-dienes **112** and azetidin-3-ones **113** yielding 1,4,7,8-tetrahydroazocin-2(3 *H* )-ones **114** (Scheme 35 ). 46


**Scheme 35** Synthesis of 1,4,7,8-tetrahydroazocin-2(3 *H* )-ones



**Scheme 36** Synthesis of azocinediones via a Rh-catalyzed cycloaddition–fragmentation process


Bower and co-workers reported a direct approach to substituted azocinediones **116** by a Rh-catalyzed cycloaddition–fragmentation process. 47 Exposure of *N* -cyclopropyl­acrylamides **115** to phosphine-ligated cationic Rh(I) catalyst systems under a CO atmosphere led to the formation of rhodacyclopentanone intermediates. The subsequent insertion of the alkene fragment into the intermediates was followed by fragmentation to give azocinediones **116** (Scheme 36 ). The overall process is considered to be equivalent to a [7+1]-cycloaddition–tautomerization sequence.

## 5 Ring-Closing Metathesis (RCM)

Another powerful method for the construction of medium-sized nitrogen-containing systems that has received considerable attention in recent years is ring-closing metathesis.

Li and co-workers reported a five-step sequence for the construction of dibenzo[ *b* , *f* ]azocinone **119** where the key step was ruthenium-mediated ring-closing metathesis. 48 Starting from methyl 4-amino-3-iodobenzoate ( **117** ) the synthesis involved Stille coupling with tributyl(vinyl)stannane, followed by acylation with 2-vinylbenzoyl chloride, Boc protection, and RCM. Using Grubbs II catalyst, RCM of compound **118** resulted in dibenzo[ *b* , *f* ]azocinone **119** (Scheme 37 ).


**Scheme 37** Synthesis of a dibenzo[ *b* , *f* ]azocinone


Starting from styryldiazoacetate **120** , azocine **122** was obtained by a sequence of two reactions: N–H insertion and RCM. 49 It was also possible to combine the carbenoid N–H insertion and RCM reactions in a one-pot procedure for the synthesis of methyl 1,2,5,6,7,8-hexahydroazocine-2-carboxylate **122** (Scheme 38 ).


**Scheme 38** Synthesis of methyl 1,2,5,6,7,8-hexahydroazocine-2-carboxylate


Carbamate **124** , obtained through condensation of aminobenzaldehyde **123** with methylamine and subsequent in situ reaction with benzyl chloroformate and then allylzinc bromide, underwent facile RCM in the presence of Grubbs II catalyst to give benzo[ *b* ]azocine **125** in 81% yield (Scheme 39 ). 38

The ring-closing metathesis approach was utilized to prepare novel 1,7-annulated azocino[3,2,1- *hi* ]indole derivatives **129** starting from indoles **126** (Scheme 40 ). 50 51 Formylation of indole **126** followed by allylation of the N-atom and condensation with nitromethane led to 1-allyl-7-(2-nitrovinyl)indole **127** . Reaction of 1-allyl-7-(2-nitrovinyl)indole **127** with allylmagnesium bromide gave 1-allyl-7-[1-(nitromethyl)but-3-enyl]indole **128** that underwent ring-closing metathesis to give azocinoindoles **129** in moderate yields.


**Scheme 39** Synthesis of a benzo[ *b* ]azocine



**Scheme 40** Synthesis of azocino[3,2,1- *hi* ]indoles


Based on combined the aza-Claisen rearrangement and ring-closing metathesis, the Majumdar group obtained pyr­imido[5,4- *b* ]azocine derivatives **135** in excellent yields (Scheme 41 ). 52 The starting 5-bromouracil derivatives **130** reacted with allylamine to give 5-allyluracils **131** , subsequent catalyzed aza-Claisen rearrangement and tosylation led to tosyl derivatives **133** . Reaction of **133** with homoallyl bromide provided the required precursor **134** for RCM using the Grubbs first-generation catalyst (Grubbs I) to give **135** .


**Scheme 41** Synthesis of pyrimido[5,4- *b* ]azocine derivatives based on combined aza-Claisen rearrangement and RCM


Using the same concept, the Majumdar group prepared RCM precursors **137** and **140** . Starting from aminonaphthalenes **136** and **139** , reaction with allylamine, subsequent aza-Claisen rearrangement, tosylation, and alkylation with homoallyl bromide gave **137** and **140** . Under the RCM conditions, naphthalene derivatives **137** and **140** gave cyclized products, naphtho[1,2- *b* ]azocines **138** and naphtho[2,1- *b* ]azocines **141** , respectively, in good yields (Scheme 42 ). 53


**Scheme 42** Synthesis of naphtho[1,2- *b* ]azocines and naphtho[2,1- *b* ]azocines


Lindsley and co-worker developed a novel six-step approach for the rapid and enantioselective synthesis of pyrrolo[1,2- *a* ]azocines **147** and **152** . 54 Commercially available aldehyde **142** was converted into ( *R* )- *N* -sulfinylaldimine **143** , followed by indium-mediated allylation yielding **144** . Subsequent alkenylation of **144** with 5-bromopent-1-ene provides **145** which underwent RCM reaction with Grubbs II catalyst to deliver **146** in 70% yield for two steps. Hydrogenation, followed by a one-pot deprotection/acetal hydrolysis/reductive amination sequence produced decahydropyr­rolo[1,2- *a* ]azocine **147** in 87% yield for two steps and with more than 98% ee (Scheme 43 ).


**Scheme 43** Synthesis of decahydropyrrolo[1,2- *a* ]azocine


Pyrrolo[1,2- *a* ]azocinone **152** was synthesized from commercial aldehyde **148** , which was converted into ( *S* )- *N* -sulfinylaldimine **149** . Indium-mediated allylation of **149** to give **150** and subsequent deprotection gave a primary amine that cyclized to give ( *S* )-5-allylpyrrolidin-2-one **151** . The alkenylation of lactone **151** with 5-bromopent-1-ene, followed by RCM with Grubbs II catalyst afforded 1,5,6,7,10,10a-hexahydropyrrolo[1,2- *a* ]azocin-3(2 *H* )-one **152** in 73% yields for two steps (Scheme 44 ).


**Scheme 44** Synthesis of 1,5,6,7,10,10a-hexahydropyrrolo[1,2- *a* ]azocin-3(2 *H* )-one


In 2013, using a modified strategy Lindsley and co-workers developed a rapid route to access pyrrolo[1,2- *a* ]-, pyrido[1,2- *a* ]-, and azepino[1,2- *a* ]azocines **153** – **155** (Scheme 45 ). 55


**Scheme 45** Synthesis of pyrrolo[1,2- *a* ]-, pyrido[1,2- *a* ]-, and azepino[1,2- *a* ]azocines


Benedetti, Penoni, and co-workers obtained benzo[ *c* ]azo­cine derivative **158** in 69% yield from enyne **157** , from **156** using a Sonogashira reaction (Scheme 46 ). 56


**Scheme 46** Synthesis of benzo[ *c* ]azocine


Hexahydroazocine **161** was formed by microwave-assisted RCM of an α-allyl-α-phenyl-α-amino acid **160** obtained in two steps from α-imino ester **159** (Scheme 47 ). 57

Dash and co-workers constructed imidazo[1,5- *a* ]azocine **163** from commercially available hydantoin **162** via a four-step procedure involving selective N-allylation and C5-alkylation and with the key step being RCM (Scheme 48 ). 58

Moss reported the efficient synthesis of a range of heterocycle-fused azocine derivatives **165** – **167** employing a directed metalation/ruthenium-catalyzed RCM approach. 59 The RCM precursors **164** were synthesized from carboxylic acids or 2-chloro-4-iodopyridine in three to four steps (Scheme 49 ).


**Scheme 47** Synthesis of hexahydroazocine


Rao and co-workers developed a common strategy for the construction of polyhydroxy azocine derivatives, including a novel example, from d -1,5-gluconolactone **168** , using an RCM protocol as the key step. 60 d -1,5-Gluconolactone **168** was converted into compound **169** by a five-step procedure. Compound **169** was subjected to allylation with allyl chloride producing *N* -allyl derivative **170** ; subsequent deprotection, oxidative cleavage, and reaction with vinylmagnesium bromide gave **171** . RCM of compound **171** afforded the cyclized products **172** and **173** . The final deprotection and hydrogenation of azocines **172** and **173** provided polyhydroxy azocine derivatives **174** and **175** in 72% and 66% yields, respectively (Scheme 50 ).


**Scheme 48** Synthesis of imidazo[1,5- *a* ]azocine



**Scheme 49** Synthesis of heterocycle-fused azocine derivatives



**Scheme 50** Synthesis of polyhydroxy azocine derivatives


Bertozzi and Sletten synthesized a novel strained azacyclooctyne **181** , which represents a new class of heterocyclic substrates for Cu-free click chemistry. 61 The synthesis involved nine steps beginning from 6-bromoglucopyranoside **176** . First, compound **176** was transformed into acyclic diene **177** via zinc reduction/reductive amination reaction followed by amide formation with methyl succinyl chloride. The eight-membered ring was constructed by RCM giving azocine **178** , which was converted into ketone **179** by oxidation and subsequent hydrogenation. The condensation of **179** with semicarbazide and then oxidation with selenium dioxide led to selenadiazole **180** . Subsequent thermal decomposition followed by saponification of the ester produced azocine **181** (Scheme 51 ).


**Scheme 51** Synthesis of a novel strained azacyclooctyne


In 2011, Danheiser and co-workers showed that the combination of ynamide-based benzannulation with RCM provides an expeditious strategy for the assembly of benzofused nitrogen heterocycles including azocines **187** – **189** . 62 The precursors **184** – **186** for RCM were obtained via benzannulation from cyclobutenones with ynamide **183** , prepared by reaction of carbamate **182** with 5-bromopent-1-en-4-yne. RCM occurred in the presence of the Grubbs II catalyst in dichloromethane (Scheme 52 ).

The synthetic use of this strategy is illustrated in its application in a concise enantioselective route to the benzoazo­cine core **190** of the antitumor agents (+)-FR900482 and (+)-FR66979. The synthesis of the benzoazocine core **191** and the completion of the formal total synthesis of FR900482 and Fr66979 are shown in Scheme 53 .

In 2013, Danheiser and co-workers employed the ‘second generation’ of benzannulation/RCM strategy, in which α-diazo ketones **192** were employed as vinylketene substrates instead of cyclobutenones. 63 Using this method hydroxy-substituted naphtho[2,3- *b* ]azocine **193** was obtained in good yield (Scheme 54 ).

In their investigations to develop concise total syntheses of some indole alkaloids possessing the azocine ring, Bennasar­ and co-workers successfully applied the combination of RCM and vinyl halide Heck cyclization for the construction of the azocinoindole moiety. The first unsuccessful attempt to work out the total synthesis of (±)-apparicine led to unexpected tetracycle **198** . 64 The required RCM substrates **195a** , **b** , prepared from 2-vinylindole-3-carbaldehyde **194** by reductive amination followed by *N* -acylation or sulfonylation, were subjected to RCM giving azocinoindoles **196** , **b** in acceptable yields. The removal of the Boc group in azocinoindole **196a** , subsequent alkylation with ( *Z* )-2-iodo­but-2-enyl tosylate and Heck cyclization yielded compound **198** possessing a bridged azocine ring (Scheme 55 ).

Changing the cyclization site from 5,6-position to 4,5-position by using as a RCM precursor 2-allyl-3-[(allylamino)methyl]indole **199** and introducing an additional isomerization step before the Heck cyclization, they succeeded in accomplishing the first total synthesis of (±)-apparicine (Scheme 56 ). 64

The combination RCM/Heck cyclization successfully was utilized for the synthesis of the upper-half of vinorelbine. 65 Reductive amination of aldehyde **200** followed by Boc-protection of the aliphatic nitrogen gave carbamate **201** , which smoothly underwent RCM in the presence of the Grubbs second-generation catalyst to give azocinoindole **202** . The sequence of *N* -Boc deprotection, alkylation with allylic bromide, *N* -indole-deprotection, and Heck cyclization gave the upper-half of vinorelbine **205** (Scheme 57 ).

RCM for the building of eight-membered nitrogen-containing cycle has also found application in the completion of the total synthesis of (–)-nakadomarin A, which shows interesting cytotoxic and antibacterial activity. A concise diastereoselective total synthesis was completed in 21 steps from d -pyroglutamic acid, wherein one of the key steps was the construction of the azocine ring via RCM (Scheme 58 ). 66

## 6 Cyclization

### 6.1 Metal-Catalyzed Cyclization

Chowdhury and co-workers described an elegant method for the synthesis of benzo[ *c* ][1,2,3]triazolo[1,5- *a* ]azocines **208** via palladium/copper-catalyzed heterocyclization. 67 The starting *ortho* -iodo azides **206** were prepared from the corresponding alcohols by mesylation and subsequent azidation. *ortho* -Iodo azides **206** underwent palladium/copper-catalyzed azide–alkyne cycloaddition with various terminal acetylenes **207** followed by arylation of the triazole to give azocine derivatives **208** . Employing 1,3-diethynylbenzene ( **209** ) as a reactant, led to bis-heteroannulation giving azocine **210** in moderate yield (Scheme 59 ). It is worth noting that the protocol included the formation of one C–C and two C–N bonds in a one-pot reaction.

Benzo[5,6]azocino[3,4- *b* ]indoles **215** were obtained in four steps from indole **211** through an intramolecular direct arylation reaction as the key step. 68 Indole **211** was first treated with amine **212** to give amide **213** ; *N* -Boc-protection or *N* -methylation gave compounds **214** . The cyclization of **214** mediated by Pd(0) delivered benzo[5,6]azocino[3,4- *b* ]indoles **215** (Scheme 60 ).


**Scheme 52** Synthesis of benzo[ *b* ]azocines



**Scheme 53** Synthesis of the benzoazocine core of (+)-FR900482 and the completion of the formal total synthesis of the antitumor agents (+)-FR900482 and (+)-FR66979; Teoc = [2-(trimethylsilyl)ethoxy]carbonyl



**Scheme 54** Synthesis of naphtho[2,3- *b* ]azocine **193**



**Scheme 55** Synthesis of a tetracyclic azocine derivative


Pyrroloazocine **219** and azocinoindoles **217** were obtained via a palladium-catalyzed norbornene-mediated tandem process involving the intramolecular *ortho* -alkylation of an aromatic C–H bond followed by intramolecular direct arylation reaction from compounds **216** and **218** , respectively (Scheme 61 ). 69


**Scheme 56** The total synthesis of (±)-apparicine


The Van der Eycken group, in 2009, developed a short and selective approach towards azocino[5,4- *b* ]indoles **221** using a microwave-assisted Hg(OTf) _2_ -catalyzed intramolecular carbocyclization of amides **220** prepared from corresponding tryptamines and 3-substituted prop-2-ynoic acids­ (Scheme 62 ). 70

In 2011, the Van der Eycken group elaborated a novel procedure for the construction of the interesting azocino[ *cd* ]indoles **224** via a Pd-catalyzed intramolecular acetylene hydroarylation. 71 The required for the cyclization, substrates **223** were synthesized by DCC-mediated amidation of suitable 4-bromotryptamines **222** and various propynoic acid derivatives. Microwave-assisted Pd-catalyzed cyclization of indoles **223** proceeded smoothly leading to regio- and stereoselective azocino[4,5,6- *cd* ]indole derivatives **224** (Scheme 63 ).

The Van der Eycken group have also reported the synthesis of azocinoindoles via an efficient gold-catalyzed post-Ugi intramolecular hydroarylation. 72 73 Ugi-adducts **225** and **227** underwent 8- *endo* - *dig* cyclization leading to azocino[5,4,3- *cd* ]indoles **226** and azocino[5,4- *b* ]indoles **228** , respectively. The merits of this method are good to excellent yields, a wide range of functional groups introduced during the Ugi reaction, and selectivity for 8- *endo* - *dig* cyclization (Scheme 64 ).


**Scheme 57** Synthesis of the upper-half of vinorelbine



**Scheme 58** The completion of the total synthesis of (–)-nakadomarin A



**Scheme 59** Synthesis of benzo[ *c* ][1,2,3]triazolo[1,5- *a* ]azocines and a bis-annulated azocine



**Scheme 60** Synthesis of benzo[5,6]azocino[3,4- *b* ]indoles; EOM = ethoxymethyl



**Scheme 61** Synthesis of dibenzo[ *c* , *f* ]pyrrolo[1,2- *a* ]azocine and dibenzo[3,4:6,7]azocino[1,2- *a* ]indoles



**Scheme 62** Synthesis of azocino[5,4- *b* ]indoles



**Scheme 63** Synthesis of azocino[4,5,6- *cd* ]indole derivatives



**Scheme 64** Synthesis of azocino[5,4,3- *cd* ]indoles and azocino[5,4- *b* ]indoles


Using a cationic gold-catalyzed intramolecular hydroarylation reaction of β-lactam-tethered allenyl indoles **229** , Alcaide, Almendos, and co-workers obtained tetrahydroazeto[1′,2′;1,2]azocino[3,4- *b* ]indoles **230** as single isomers in good yields (Scheme 65 ). 74 The formation of azocines **230** was rationalized through an 8- *endo* carbocyclization of the indole group towards the terminal allene carbon. The gold-catalyzed cyclization allowed the regioselective formation of fused β-lactams without harming the sensitive four-membered heterocycle.


**Scheme 65** Synthesis of tetrahydroazeto[1′,2′;1,2]azocino[3,4- *b* ]indoles


Pentacyclic azocine derivative **232** was generated in good yield via a novel gold-catalyzed cascade cyclization from *N* -[(2-azidophenyl)ethynyl]benzamides **231** (Scheme 66 ). 75

Echavarren and co-workers reported the synthesis of the azocino[5,4- *b* ]indole core skeleton of the lundurines by gold-catalyzed 8- *endo* - *dig* cyclization of an alkynylindole. 76 The AuCl _3_ -catalyzed cyclization of 2-[2-(2-ethynyl-5-oxopyrrolidino)ethyl]indole **233** , prepared in seven steps from 2-(1 *H* -indol-3-yl)acetate, afforded azocinoindole **234** in 55% isolated yield (Scheme 67 ); the feasibility of using other gold complexes was considered.


**Scheme 66** Synthesis of a pentacyclic azocine derivative



**Scheme 67** Synthesis of the azocino[5,4- *b* ]indole core skeleton of the lundurines


### 6.2 Radical Cyclization

The Majumdar group developed a new efficient method for the synthesis of pyrimidoazocine derivatives **238** via the first example of an 8- *endo* - *trig* thiophenol-mediated radical cyclization. 77 The radical precursors **237** were prepared from pyrimidines **235** , products of the aza-Claisen rearrangement of *N* -allyl-substituted pyrimidines, by tosylation and the subsequent reaction of intermediates **236** with propargyl bromide. The alkenyl radicals were generated from thiophenol initiated by benzoyl peroxide. The pyrimido[5,4- *b* ]azocines **237** were obtained in excellent yields (Scheme 68 ).


**Scheme 68** Synthesis of pyrimido[5,4- *b* ]azocines


On developing the total synthesis of (±)-apparacine, Bennasar and co-workers prepared azocino[4,3- *b* ]indole **242** via radical cyclization. 64 The synthesis of compounds **242** began with the preparation of selenoester **241** as the radical precursor. Selenoester **241** was prepared by reductive amination of aldehyde **239** , followed by Boc protection of the resulting secondary amine, and phenylselenenation of the corresponding carboxylic acid. The treatment of selenoester **241** with Bu _3_ SnH as the radical mediate and Et _3_ B as the initiator led to the formation of azocinoindole **242** . The reaction of **242** with methyllithium followed by dehydration of the intermediate tertiary alcohol provided azocinoindole **243** (Scheme 69 ), which successfully used in the synthesis of (±)-apparacine (see Scheme 55 ).


**Scheme 69** Synthesis an azocino[4,3- *b* ]indole


In their investigations, Li and co-workers generated azocine derivatives **245** and **247** starting from diesters **244** and **246** by a sequence of reactions in which the azocine-formation step was radical cyclization. 78 In the case of azocine **245** it was a Mn(III)-mediated oxidative radical process, whereas the azocine system **247** was obtained by a reductive radical process (Scheme 70 ).


**Scheme 70** Synthesis of bridged azocine derivatives


Diaba, Bonjoch, and co-worker obtained morphan compounds **249** via the first intramolecular atom transfer radical process between trichloroacetamide and enol acetate used as a radical acceptor. 79 The reaction was promoted by Grubbs II catalyst, thus expending the scope of these catalysts beyond the metathesis reaction (Scheme 71 ).

This reaction enabled the construction of the tricyclic skeleton of the immunosuppressant FR901483. The required proradical trichloroacetamide **251** was synthesized in five steps starting from azaspirodecane **250** . The treatment of ketone **251** with isopropenyl acetate gave a regioisomeric mixture of enol acetate **252** in a 1.8:1 ratio, the unseparated mixture was treated with Grubbs II catalyst affording the diazatricyclic derivative **253** , its epimer **254** , and unexpected mixture of enones **255** . The reaction of compound **253** with zinc led to dechlorinated derivative **256** , possessing the tricyclic skeleton of immunosuppressant FR901483, in 58% yield (Scheme 72 ). 79


**Scheme 71** Synthesis of morphan compounds



**Scheme 72** Synthesis of tricyclic skeleton of immunosuppressant FR901483


Li and co-workers used a route based on the iodine-atom-transfer radical 8- *endo* cyclization to synthesize a number of azocine derivatives. 80 Thus, *N* -acyloxazolidin­ones **257** underwent 8- *endo* cyclization promoted by BF _3_ ·OEt _2_ /H _2_ O leading to the formation of oxazoloazocine **258** in high yields with excellent regio- and stereoselectivity (Scheme 73 ). It is interesting to note that the product configuration was changed from 3,8- *trans* to 3,8- *cis* .

Li and co-workers also showed that in the presence of Mg(ClO _4_ ) _2_ and a bis(oxazoline) ligand, *N* -ethoxycarbonyl-substituted 2-iodo- *N* -(pent-4-enyl)alkanamides **259** underwent 8- *endo* cyclization giving only 3,5- *trans* -substituted azocan-2-ones **260** in excellent yields (Scheme 74 ).


**Scheme 73** Synthesis of oxazoloazocines



**Scheme 74** Synthesis of azocan-2-ones


Similarly, the BF _3_ ·OEt _2_ /H _2_ O promote reaction of *N* -(2-allylaryl)- *N* -(ethoxycarbonyl)-2-iodoalkanamides **261** afforded benzo[ *b* ]azocines **262** with *cis* -3,5-configuration in high yields (Scheme 75 ).


**Scheme 75** Synthesis of benzo[ *b* ]azocines


### 6.3 Friedel–Crafts Cyclization

Azocino[3,4- *b* ]indol-1-ones **264** were obtained via acid-catalyzed intramolecular Friedel–Crafts cyclization of 1-methyl- *N* -(4-oxobutyl)indole-2-carboxamides **263** in low yields (Scheme 76 ). 81


**Scheme 76** Synthesis of azocino[3,4- *b* ]indol-1-ones via acid-catalyzed intramolecular Friedel–Crafts cyclization


Pandey and co-workers developed a general route for the synthesis of azocino[5,4- *b* ]indoles **269** starting from allyl bromide **265** which prepared from Morita–Baylis–Hillman­ adducts. 82 The reaction of tryptamine with allyl bromide **265** gives **266** , protection of the nitrogen atoms in **266** gives **267** , and saponification of the ester group in **267** gave indoles **268** which underwent Friedel–Crafts intramolecular cyclization affording azocino[5,4- *b* ]indoles **269** in good yields (Scheme 77 ).


**Scheme 77** Synthesis of azocino[5,4- *b* ]indoles


Kim and Seo used the Friedel–Crafts reaction as the key step for the construction of azocine ring in the tetracyclic compound **274** . 83 Aminoacrylate **271** , prepared from 3,4-dimethoxyphenethylamine via amidation reaction with 2-iodobenzoic acid and subsequent Michael addition of ethyl propynoate, was subjected to the Heck reaction giving isoindole **272** . The hydrogenation of isoindole **272** followed by hydrolysis provide derivative **273** which by the action of polyphosphoric acid was converted into tetracyclic compound **274** (Scheme 78 ).


**Scheme 78** Synthesis of benzo[5,6]azocino[2,1- *a* ]isoindole


The same sequence of reactions was used in the case of the synthesis of an azocine alkaloid, magallanesine (Scheme 79 ). 83


**Scheme 79** Synthesis of magallanesine


### 6.4 Other Examples of Cyclization

In a new synthetic route for the synthesis of the dasycarpidone skeleton and for the total synthesis of (±)-uleine, Patir and Uludag used acid-catalyzed intramolecular cyclization to construct the azocino[4,3- *b* ]indole core. 84 Reduction of ketoamide **275** with borane–dimethyl sulfide complex and the subsequent acidification of the resulted alcohol **276** with TFA led to **277** , which was treated in situ with DDQ furnishing the desired tetracyclic compounds **278** in good yield. Four further steps were required to complete the total synthesis of (±)-uleine from azocinoindole **278** (Scheme 80 ).


**Scheme 80** Synthesis of a tetracyclic azocine system with the dasycarpidone skeleton


Azocinoindoles **280** and **283** were obtained by Hamada and co-workers via a novel acid-promoted skeletal rearrangement of 2- or 3-alkylideneindolenium cations generated from compounds **279** and **282** . 85 86 A reaction cascade leading to the azocine system involved intramolecular *ipso* -Friedel–Crafts alkylation of phenols, rearomatization of the spirocyclohexadienone unit, and iso-Pictet–Spengler reaction. In addition to the targeted azocinoindoles, pyrrolidine derivatives **281** and **284** were isolated as the major byproducts (Scheme 81 ).


**Scheme 81** Acid-promoted skeletal rearrangement of 2- or 3-alkylidene­indolenium cations


Azocino[4,3- *b* ]indoles **287** were synthesized by the oxidative cyclization of [3-(3-oxopiperidin-2-yl)indol-2-yl]malonates **286** obtained in four steps from dimethyl or di- *tert* -butyl malonates **285** . 87 Furthermore, these azocino[4,3- *b* ]indoles were successfully used for the total synthesis of (±)- and (–)-actinophyllic acid (Scheme 82 ).


**Scheme 82** Synthesis of azocino[4,3- *b* ]indoles


Reitz and co-workers have prepared benzo[ *d* ]azocines **294** and **295** which are formally analogues of Phe-Ala or Ala-Phe dipeptides joined together on their side chains. 88 The synthesis began with the conversion of the *N* -Boc-protected 2′-iodo- l -phenylalanine **288** into *N* -Fmoc derivative **289** . Negishi coupling of **289** with either Boc-(β-I)- d -Ala-OMe or Boc-(β-I)- l -Ala-OMe gave dipeptides **290** and **291** , respectively. Removals of Boc- and benzyl ester groups of bis-amino acids **290** and **291** and amide formation and cyclization with 1-hydroxy-7-azabenzotriazole (HOAt) and *O* -(7-azabenzotriazole-1-yl)- *N* , *N* , *N* ′, *N* ′-tetramethyluronium hexa­fluorophosphate (HATU) provided azocines **292** and **293** . Fmoc-deprotection followed by simultaneous amidolysis of the ester with methylamine and acetylation led to dipeptides **294** and **295** (Scheme 83 ).


**Scheme 83** Synthesis of benzo[ *d* ]azocines; NEM = *N* -ethylmorpholine



**Scheme 84** Synthesis of hexahydrochromeno[3,4- *c* ]azocine; HMTA = hexamethylenetetramine


Hexahydrochromeno[3,4- *c* ]azocine **297** was obtained from chromene **296** by cyclization which occurred during Duff formylation, but the yield of the cyclic product was poor at only 9% (Scheme 84 ). 89

## 7 Microwave- and Photo-Assisted Reactions

Alcaide, Almendos, and co-workers developed a method for the synthesis of structurally novel bicyclic azocine-fused β-lactams **299** and **300** in the absence of any metal catalyst. 90 This was the first example of metal-free preparation of eight-membered rings by the thermolysis of nonconjugated azetidin-2-one-tethered bis(allenes) **298** on application of microwave irradiation. Azocines **299** and **300** were isolated as single regio- and diastereoisomers (Scheme 85 ).


**Scheme 85** Synthesis of novel bicyclic azocine-fused β-lactams


The Van der Eycken group elaborated a new approach towards the construction of 5,6,7,8-tetrahydrodibenzo[ *c* , *e* ]azocines **303** via a microwave-assisted copper-catalyzed intramolecular A ^3^ -coupling reaction. 91 Formed in situ by Boc deprotection of biaryl compounds **301** , biaryl derivatives **302** with both amino and aldehyde groups reacted with the suitable alkynes in the presence of CuBr under focused microwave irradiation thus forming dibenzo[ *c* , *e* ]azocines **303** in good to excellent yields (Scheme 86 ).


**Scheme 86** Synthesis of 5,6,7,8-tetrahydrodibenzo[ *c* , *e* ]azocines


Yudin and Cheung found that *N* -vinyl-β-lactams **304** underwent microwave-assisted ring-expansion, resulting from [3,3]-sigmatropic rearrangement between two strategically placed alkene moieties on the β-lactam, giving azocines **305** in yields of 8–86% (Scheme 87 ). 92 93


**Scheme 87** [3,3]-Sigmatropic rearrangement of *N* -vinyl-β-lactams


On irradiation of 4-diazo-4 *H* -imidazole **306** in hexafluorobenzene gave the unusual imidazo[3,4- *a* ]azocine **307** in 51% yield (Scheme 88 ). 94


**Scheme 88** Synthesis of imidazo[3,4- *a* ]azocine


Kutateladze and co-workers synthesized epoxybenzoazo­cines **309** and **311** from aniline derivatives **308** and **310** , respectively, by photo-generation of azaxylylenes and their subsequent intramolecular [4+4] cycloaddition with a furan-containing pendant tethered either via the aniline nitrogen or through the carbonyl-group-containing fragment (Scheme 89 ). 95


**Scheme 89** Synthesis of epoxybenzoazocines


## 8 Other Methods

### 8.1 Cascade and Tandem Reactions

Nakamura and co-workers elaborated an efficient synthesis of azocine derivatives **313** from *O* -propargylic oximes **312** in good to excellent yields by the means of a Rh-catalyzed 2,3-rearrangement/heterocyclization cascade sequence. 96 It is noteworthy that the chirality of the substrate was maintained throughout the cascade process to afford optically active azocines **313d** (Scheme 90 ).


**Scheme 90** Synthesis of azocine derivatives


She, Xie, and co-workers accomplished a gold-catalyzed 1,2-acyloxy migration/intramolecular [3+2]-cycloaddition cascade reaction for the construction of unsaturated azocines **315** starting from enynyl esters **314** (Scheme 91 ). 97


**Scheme 91** A gold-catalyzed 1,2-acyloxy migration/intramolecular [3+2]-cycloaddition cascade reaction


In 2016, She, Xie, and co-workers subsequently expanded this gold-catalyzed 1,2-acyloxy migration/intramolecular [3+2]-cycloaddition cascade reaction to the synthesis of benzo[ *d* ]azocines **317** from 1,9-enynyl esters **316** . The reaction proceeded under mild conditions leading to benzoazocines **317** in good to excellent yields of 55–82% (Scheme 92 ). 98


**Scheme 92** Synthesis of benzo[ *d* ]azocines **317**


Kumar and co-workers synthesized benzo[ *b* ]azocines **318** through an unprecedented one-pot, triflic acid mediated, tandem Michael addition–Fries rearrangement of sorbyl­anilides **319** . 99 The reaction is proposed to proceed via a δ-lactam intermediate, earlier considered unreactive for the Fries rearrangement (Scheme 93 ).

In their research concerning the total synthesis of (±)-actinophyllic acid, Martin and co-workers constructed the tetracyclic core of the natural compound in a single chemical operation via a novel Lewis acid catalyzed cascade of reactions involving stabilized carbocations and π-nucleophiles. 100 The treatment of a mixture of electrophile precursor indole **320** and π-nucleophiles **321** with TMSOTf in the presence of 2,6-di- *tert* -butylpyridine, followed by addition of NaOMe in MeOH at –78 °C gave tetracyclic systems **322** in 53–80% yields (Scheme 94 ).


**Scheme 93** Synthesis of benzo[ *b* ]azocines via tandem Michael addition–Fries rearrangement



**Scheme 94** Synthesis of tetracyclic azocinoindoles


### 8.2 Aldol Condensation

In their work on the total syntheses of (–)-FR901483 and (+)-8- *epi* -FR901483, Huang and co-workers successfully used the aldol condensation for the construction of the azocine ring. 101 102 Starting from the known chiron ( *R* )-1-allyl-3-benzyloxypiperidine-2,5-dione, piperidin-3-ol **324** was obtained in four steps. The oxidation of **323** gave a ketone that underwent an intramolecular aldol ring-closure reaction forming azocine derivative **324** (Scheme 95 ).


**Scheme 95** Synthesis of bridged azocine derivatives


Uludag and co-workers ring-closed 1-oxo-1,2,3,4-tetrahydrocarbazole **325** by a NaH-promoted intramolecular aldol condensation to give the azocino[4,3- *b* ]indole system **326** (Scheme 96 ). 103

### 8.3 Thermolysis

Thermolysis of hydrazine **327** in *m* -xylene under reflux led to the impressive cyclopropa[3,4]azocino[1,2- *a* ]benz­imidazole **328** in a poor yield of 11% with the two other products **329** and **330** in higher yields of 22% and 33% (Scheme 97 ). 104


**Scheme 96** Synthesis of a bridged azocino[4,3- *b* ]indole



**Scheme 97** Synthesis of cyclopropa[3,4]azocino[1,2- *a* ]benzimidazole


Thermolysis of the 12-membered ring aza-enediyne **331** in benzene in the presence of catalytic amounts of *p* -toluenesulfonic acid produced the addition-dehydration product, naphtho[2,3- *b* ]azocine **332** in 18% yield, however, the major product of this reaction was *N* -tosyl lactam **333** in 28% yield (Scheme 98 ). 105


**Scheme 98** Thermolysis of a 12-membered ring aza-enediyne


### 8.4 Ring Opening

Tetracyclic azocine derivative **336** possessing a paullone-like structural framework was obtained in a single step from a novel 2,2′-spirobi[indolin]-3-one **335** prepared by Cu-mediated intramolecular cascade reaction of cyclopenta[ *b* ]indole **334** . 106 Compound **335** when treated with methanolic KOH underwent demesylation followed by ring opening and subsequent aromatization to give **336** in 90% yield (Scheme 99 ).


**Scheme 99** Synthesis of a tetracyclic azocine derivative


Yavari and Seyfi found that furo[2′,3′:2,3]cyclopenta[1,2- *b* ]pyrroles **338** , obtained by Wittig reaction from oxo­indeno[1,2- *b* ]pyrroles **337** and DMAD, underwent Et _3_ N-mediated ring opening thus affording tetrahydrobenzo[ *c* ]furo[3,2- *e* ]azocines **339** in good yields (Scheme 100 ). 107


**Scheme 100** Synthesis of tetrahydrobenzo[ *c* ]furo[3,2- *e* ]azocines


### 8.5 Other Methods

Waghmode and co-workers synthesized epoxy-bridged benzo[ *d* ]azocines **342** in good to excellent yields from 1-(bromomethyl)-3-(tosyloxy)chromane **340** via nucleophilic substitution with various benzylamine derivatives **341** (Scheme 101 ). 108


**Scheme 101** Synthesis of epoxy-bridged benzoazocines via nucleo­philic substitution


Jia and co-workers elaborated a highly enantioselective palladium/ l -proline-catalyzed α-arylative desymmetrization of cyclohexanones **343** leading to a series of optically active morphan derivatives **344** with α-carbonyl tertiary stereocenters in good yields (Scheme 102 ). 109


**Scheme 102** Synthesis of a series of optically active morphan derivatives


Xu, Li, and co-workers have designed and synthesized novel neonicotinoid analogues **346** – **348** with an aza-bridged azocine fragment. 110 Azocine derivatives **346** – **348** were prepared by reaction of imidazole **345** with glutaraldehyde and a primary amine hydrochloride (aliphatic amines, phenylhydrazines, and anilines) (Scheme 103 ).


**Scheme 103** Synthesis of novel neonicotinoid analogues


Azocine derivative **350** was obtained in 16% yield via cationic aza-Cope rearrangement of aminoketal **349** (Scheme 104 ). 111

Yao, Wu, and co-workers developed a novel facile and efficient route for the synthesis of benzo[ *b* ]naphtha[2,3- *d* ]azocinones **353** through a palladium-catalyzed reaction of 2-alkynylanilines **351** with 2-(2-bromobenzylidene)cyclobutanones **352** . 112 During the reaction process, double carbometalation resulting in the formation of three new bonds was involved (Scheme 105 ).

Boeckman and co-workers obtained azocine derivatives **355** and **357** using a one-pot, aza-Wittig/retro-aza-Claisen sequence from 2-vinylcyclobutanecarbaldehydes **354** and **356** , respectively. 113 The rearrangement sequence proceeded under mild conditions affording azocines **355** and **357** in 75–92% yields (Scheme 106 ).

Modification of a previously reported procedure 114 allowed Raffa and co-workers obtain the polycyclic system, 5,7:7,12-dimethanopyrazolo[3,4- *b* ]pyrazolo[3′,4′:2,3]azepino[4,5- *f* ]azocine **360** . 115 Methylaminopyrazoles **358** and hexane-2,5-dione ( **359** ) reacted in refluxing 1,4-dioxane in the presence of *p* -toluenesulfonic acid thus leading to compounds **360** in 10–37% yields (Scheme 107 ).

Systematically investigating the reactivity of the palladacycles obtained in their studies, Vicente, Saura-Llamas, and co-workers synthesized various azocine-containing systems. Thus, heating of complex **361** in the presence of TlOTf and 2,4-dimethylphenyl isocyanide gave azocine **363** through insertion of isocyanide and C–N coupling process (Scheme 108 ). 116

The treatment of eight-membered palladacycles **364** and palladacycles **366** with CO afforded benzo[ *d* ]azocine-2,4-(1 *H* ,3 *H* )-diones **365** 117 or hexahydrobenzo[ *d* ]azocin­ones **367** , 118 which resulted from the insertion of a molecule of CO into the Pd–C bond and subsequent C–N reductive coupling (Scheme 109 ).

These methods were extended to the preparation of dibenzo[ *c* , *e* ]azocines **369** / **370** and **371** via insertion of CO and isocyanide, respectively (Scheme 110 ). 119

## 9 Conclusion

In recent years, many new pathways towards eight-membered azaheterocycles have been elaborated including domino approaches, MCRs, metal-catalyzed cyclizations, RCM, and ring-expansion strategies. These approaches provide environmentally friendly and step-economical access towards several annulated azocines with substantial biological activity and natural compounds. However, much work remains to be done to elaborate general synthetic strategy towards medium-sized nitrogen heterocycles including azo­cines.


**Scheme 104** Cationic aza-Cope rearrangement of an aminoketal



**Scheme 105** Synthesis of benzo[ *b* ]naphtha[2,3- *d* ]azocinones



**Scheme 106** Synthesis of azocine derivatives



**Scheme 107** Synthesis of 5,7:7,12-dimethanopyrazolo[3,4- *b* ]pyrazolo[3′,4′:2,3]azepino[4,5- *f* ]azocine



**Scheme 109** Synthesis of benzo[ *d* ]azocinediones or hexahydrobenzo[ *d* ]azocinones



**Scheme 108** Synthesis of iminobenzo[ *d* ]azocine



**Scheme 110** Synthesis of dibenzo[ *c* , *e* ]azocines


## References

[1] Voskressensky L G Kulikova L N Borisova T N Varlamov A V Adv. Heterocycl. Chem. 2008 96 81

[2] Rössle M Christoffers J Synlett 2006 106

[3] Behler F Habecker F Saak W Klüner T Christoffers J Eur. J. Org. Chem. 2011 4231

[4] Penning M Christoffers J Eur. J. Org. Chem. 2014 2140

[5] Penning M Aeissen E Christoffers J Synthesis 2015 47 1007

[6] Varlamov A V Borisova T N Voskressensky L G Soklakova T A Kulikova L N Chernyshov A A Alexandrov G G Tetrahedron Lett. 2002 43 6767

[7] Voskressensky L G Borisova T N Ovcharov M V Kulikova L N Sorokina E A Borisov R S Varlamov A V Chem. Heterocycl. Compd. 2008 44 1510

[8] Voskressensky L G Borisova T N Ovcharov M V Sorokina E A Khrustalev V N Varlamov A V Russ. Chem. Bull. 2012 61 1603

[9] Voskressensky L G Kovaleva S A Borisova T N Listratova A V Eresko A B Tolkunov V S Tolkunov S V Varlamov A V Tetrahedron 2010 66 9421

[10] Voskressensky L G Ovcharov M V Borisova T N Kulikova L N Listratova A V Borisov R S Varlamov A V Chem. Heterocycl. Compd. 2011 47 222

[11] Voskressensky L G Ovcharov M V Borisova T N Listratova A V Kulikova L N Sorokina E A Gromov S P Varlamov A V Chem. Heterocycl. Compd. 2013 49 1180

[12] Voskressensky L G Kovaleva S A Borisova T N Eresko A B Tolkunov V S Tolkunov S V Listratova A V Candia M Altomare C Varlamov A V Chem. Heterocycl. Compd. 2013 49 930

[13] Voskressensky L G Borisova T N Chervyakova T M Matveeva M D Galaktionova D V Tolkunov S V Tolkunova V S Eresko A B Varlamov A V Chem. Heterocycl. Compd. 2014 50 1338

[14] Voskressensky L G Borisova T N Chervyakova T M Titov A A Kozlov A V Sorokina E A Samavati R Varlamov A V Chem. Heterocycl. Compd. 2014 50 658

[15] Voskressensky L G Titov A A Kobzev M S Samavati R Borisov R S Kulikova L N Varlamov A V Chem. Heterocycl. Compd. 2016 52 68

[16] Voskressensky L G Borisova T N Kovaleva S A Listratova A V Kulikova L N Khrustalev V N Ovcharov M V Varlamov A V Russ. Chem. Bull. 2012 61 370

[17] Voskressensky L G Kovaleva S A Borisova T N Titov A A Toze F Listratova A V Khrustalev V N Varlamov A V Chem. Heterocycl. Compd. 2015 51 17

[19] Voskressensky L G Listratova A V Borisova T N Kovaleva S A Borisov R S Varlamov A V Tetrahedron 2008 64 10443

[20] Voskressensky L G Kulikova L N Gozun S V Khrustalev V N Borisova T N Listratova A V Ovcharov M V Varlamov A V Tetrahedron Lett. 2011 52 4189

[21] Voskressensky L G Borisov R S Kulikova L N Kleimenov A V Borisova T N Varlamov A V Chem. Heterocycl. Compd. 2012 48 680

[22] Cho H Iwama Y Sugimoto K Kwon E Tokuyama H Heterocycles 2009 78 1183

[23] Cho H Iwama Y Sugimoto K Mori S Tokuyama H J. Org. Chem. 2010 75 627 20039606 10.1021/jo902177p

[24] Cho H Tetrahedron 2014 70 3527

[25] Cho H Iwama Y Okano K Tokuyama H Synlett 2013 24 813

[26] González-Gómez Á Domónguez G Pérez-Castells J Tetrahedron 2009 65 3378

[27] Zhang L Chang L Hu H Wang H Yao Z.-J Wang S Chem. Eur. J. 2014 20 2925 24519999 10.1002/chem.201304524

[28] Harthong S Bach R Besnard C Guénée L Lacour J Synthesis 2013 45 2070

[29] Mohammadizadeh M R Alborz M Tetrahedron Lett. 2014 55 6808

[30] Malkova A V Polyanskii K B Soldatenkov A T Soldatova S A Merkulova N L Khrustalev V N Russ. J. Org. Chem. 2016 52 219

[31] Majumdar K C Chattopadhyay B Samanta S Tetrahedron Lett. 2009 50 3178

[32] Majumdar K C Samanta S Chattopadhyay B Tetrahedron Lett. 2009 50 4866

[33] Majumdar K C Samanta S Chattopadhyay B Nandi R K Synthesis 2010 985

[34] Majumdar K C Mondal S Ghosh D Chattopadhyay B Synthesis 2010 1315

[35] Majumdar K C Ghosh T Chakravorty S Tetrahedron Lett. 2010 51 3372

[36] Lee H S Kim S H Kim T H Kim J N Tetrahedron Lett. 2008 49 1773

[37] Lee H S Kim S H Gowrisankar S Kim J N Tetrahedron 2008 64 7183

[38] Sunderhaus J D Dockendorff C Martin S F Tetrahedron 2009 65 6454 20625454 10.1016/j.tet.2009.05.009 PMC2897759

[39] Bennasar M.-L Zulaica E Solé D Alonso S Tetrahedron 2012 68 4641

[40] Sahn J J Martin S F Tetrahedron Lett. 2011 52 6855 22711939 10.1016/j.tetlet.2011.10.022 PMC3375694

[41] Jäger M Görls H Günther W Schubert U S Chem. Eur. J. 2013 19 2150 23255476 10.1002/chem.201202790

[42] Peshkov A A Peshkov V A Pereshivko O P Van Hecke K Kumar R Van der Eycken E V J. Org. Chem. 2015 80 6598 25996648 10.1021/acs.joc.5b00670

[43] Yu R T Friedman R K Rovis T J. Am. Chem. Soc. 2009 131 13250 19711950 10.1021/ja906641d PMC2774260

[44] Koya S Yamanoi K Yamasaki R Azumaya I Masu H Saito S Org. Lett. 2009 11 5438 19904923 10.1021/ol902299p

[45] Kumar P Zhang K Louie J Angew. Chem. Int. Ed. 2012 51 8602 10.1002/anie.201203521 PMC3557805 22806996

[46] Thakur A Facer M E Louie J Angew. Chem. Int. Ed. 2013 52 12161 10.1002/anie.201306869 PMC4113093 24573793

[47] Shaw M H Croft R A Whittingham W G Bower J F J. Am. Chem. Soc. 2015 137 8054 26090897 10.1021/jacs.5b05215 PMC4508204

[48] Hassam M More S V Li W.-S Mendeleev Commun. 2014 24 159

[49] Pavlyuk O Teller H McMills M C Tetrahedron Lett. 2009 50 2716

[50] Wood K Black D St. C Namboothiri I N. N Kumar N Tetrahedron Lett. 2010 51 1606

[51] Wood K Black D St. C Kumar N Tetrahedron 2011 67 4093

[52] Majumdar K C Mondal S Ghosh D Synthesis 2010 1176

[53] Majumdar K C Samanta S Chattopadhyay B Nandi R K Synthesis 2010 863

[54] Fadeyi O O Senter T J Hahn K N Lindsley C W Chem. Eur. J. 2012 18 5826 22473565 10.1002/chem.201200629 PMC3540812

[55] Senter T J Schulte M L Konkol L C Wadzinski T E Lindsley C W Tetrahedron Lett. 2013 54 1645 23459400 10.1016/j.tetlet.2013.01.041 PMC3580858

[56] Benedetti E Lomazzi M Tibiletti F Goddard J.-P Fensterbank L Malacria M Palmisano G Penoni A Synthesis 2012 44 3523

[57] Curto J M Kozlowski M C J. Org. Chem. 2014 79 5359 24828423 10.1021/jo500707t PMC4059215

[58] Dhara K Midya G C Dash J J. Org. Chem. 2012 77 8071 22938501 10.1021/jo301234r

[59] Moss T A Tetrahedron Lett. 2013 54 4254

[60] Jagadeesh Y Ramakrishna K Rao B V Tetrahedron: Asymmetry 2012 23 697

[61] Sletten E M Bertozzi C R Org. Lett. 2008 10 3097 18549231 10.1021/ol801141k PMC2664610

[62] Mak X Y Crombie A L Danheiser R L J. Org. Chem. 2011 76 1852 21322545 10.1021/jo2000308 PMC3062847

[63] Willumstad T P Haze O Mak X Y Lam T Y Wang Y.-P Danheiser R L J. Org. Chem. 2013 78 11450 24116731 10.1021/jo402010b PMC3861882

[64] Bennasar M.-L Zulaica E Solé D Roca T García-Díaz D Alonso S J. Org. Chem. 2009 74 8359 19824689 10.1021/jo901986v

[65] Bennasar M.-L Solé D Zulaica E Alonso S Tetrahedron 2013 69 2534

[66] Nilson M G Funk R L Org. Lett. 2010 12 4912 20932022 10.1021/ol102079z PMC3151009

[67] Brahma K Achari B Chowdhury C Synthesis 2013 45 545

[68] Putey A Popowycz F Do Q.-T Bernard P Talapatra S K Kozielski F Galmarini C M Joseph B J. Med. Chem. 2009 52 5916 19743863 10.1021/jm900476c

[69] Blaszykowski C Aktoudianakis E Alberico D Bressy C Hulcoop D G Jafarpour F Joushaghani A Laleu B Lautens M J. Org. Chem. 2008 73 1888 18260672 10.1021/jo702052b

[70] Donets P A Van Hecke K Van Meervelt L E Van der Eycken V Org. Lett. 2009 11 3618 19610606 10.1021/ol901356h

[71] Peshkov V A Van Hove S Donets P A Pereshivko O P Van Hecke K Van Meervelt L Van der Eycken E V Eur. J. Org. Chem. 2011 1837

[72] Kumar A Li Z Sharma S K Parmar V S Van der Eycken E V Chem. Commun. 2013 49 6803 10.1039/c3cc42704h 23783807

[73] Modha S G Vachhani D D Jacobs J Van Meervelt L Van der Eycken E V Chem. Commun. 2012 48 6550 10.1039/c2cc32586a 22622200

[74] Alcaide B Almendros P Cembellín S del Campo T M J. Org. Chem. 2015 80 4650 25880256 10.1021/acs.joc.5b00535

[75] Tokimizu Y Oishi S Fujii N Ohno H Org. Lett. 2014 16 3138 24874723 10.1021/ol5012604

[76] Ferrer C Escribano-Cuesta A Echavarren A M Tetrahedron 2009 65 9015

[77] Majumdar K C Mondal S Ghosh D Tetrahedron Lett. 2010 51 348

[78] Zhang J Wang Y.-Q Wang X.-W Li W.-D Z J. Org. Chem. 2013 78 6154 23724931 10.1021/jo4007943

[79] Diaba F Martínez-Laporta A Bonjoch J J. Org. Chem. 2014 79 9365 25203675 10.1021/jo501110c

[80] Fang X Liu K Li C J. Am. Chem. Soc. 2010 132 2274 20121130 10.1021/ja9082649

[81] Cincinelli R Dallavalle S Merlini L Nannei R Scaglioni L Tetrahedron 2009 65 3465

[82] Pandey G Kant R Batra S Tetrahedron Lett. 2015 56 930

[83] Seo S.-Y Kim G Tetrahedron Lett. 2015 56 3835

[84] Patir S Uludag N Tetrahedron 2009 65 115

[85] Yokosaka T Nemoto T Hamada Y Chem. Commun. 2012 48 5431 10.1039/c2cc31699d 22543858

[86] Yokosaka T Kanehira T Nakayama H Nemoto T Hamada Y Tetrahedron 2014 70 2151

[87] Martin C L Overman L E Rohde J M J. Am. Chem. Soc. 2010 132 4894 20218696 10.1021/ja100178u PMC2851836

[88] Falcon B V Creighton C J Parker M H Reitz A B Synth. Commun. 2008 38 411

[89] Ranatunga S Tang C.-H A Kang C W Kriss C L Kloppenburg B J Hu C.-C A Del Valle J R J. Med. Chem. 2014 57 4289 24749861 10.1021/jm5002452 PMC4032190

[90] Alcaide B Almendros P Aragoncillo C Fernandez I Gomez-Campillos G J. Org. Chem. 2014 79 7075 25010752 10.1021/jo501231q

[91] Bariwal J B Ermolat’ev D S Glasnov T N Van Hecke K Mehta V P Van Meervelt L Kappe C O Van der Eycken E V Org. Lett. 2010 12 2774 20481446 10.1021/ol1008729

[92] Cheung L L. W Yudin A K Org. Lett. 2009 11 1281 19239262 10.1021/ol900118d

[93] Cheung L L. W Yudin A K Chem. Eur. J. 2010 16 4100 20186912 10.1002/chem.200902748

[94] Smith M R Blake A J Hayes C J Stevens M F. G Moody C J J. Org. Chem. 2009 74 9372 19921793 10.1021/jo902165w

[95] Mukhina O A Kumar N N. B Cowge T M Kutateladze A G J. Org. Chem. 2014 79 10956 25370821 10.1021/jo5019848 PMC4242042

[96] Nakamura I Sato Y Takeda K Terada M Chem. Eur. J. 2014 20 10214 25044489 10.1002/chem.201403637

[97] Zhao C Xie X Duan S Li H Fang R She X Angew. Chem. Int. Ed. 2014 53 10789 10.1002/anie.201406486 25111560

[98] Feng S Wang Z Zhang W Xie X She X Chem. Asian J. 2016 11 2167 27310714 10.1002/asia.201600593

[99] Anand A Singh P Mehra V Kumar V Mahajan M P Tetrahedron Lett. 2012 53 2417

[100] Granger B A Jewett I T Butler J D Martin S F Tetrahedron 2014 70 4094 24882888 10.1016/j.tet.2014.03.034 PMC4034156

[101] Huo H.-H Zhang H.-K Xia X.-E Huang P.-Q Org. Lett. 2012 14 4834 22937903 10.1021/ol302165d

[102] Huo H.-H Xia X.-E Zhang H.-K Huang P.-Q J. Org. Chem. 2013 78 455 23214918 10.1021/jo302362b

[103] Uludag N Yılmaz R Asutay O Colak N Chem. Heterocycl. Compd. 2016 52 196

[104] Hehir S O’Donovan L Carty M P Aldabbagh F Tetrahedron 2008 64 4196

[105] Poloukhtine A Rassadin V Kuzmin A Popik V V J. Org. Chem. 2010 75 5953 20684502 10.1021/jo101238x

[106] Prasad B Adepu R Sharma A K Pal M Chem. Commun. 2015 51 1259 10.1039/c4cc08911a 25476865

[107] Yavari I Seyfi S Synlett 2012 23 1209

[108] Patil V P Ghosh A Sonavane U Joshi R Sawant R Jadhav S Waghmode S B Tetrahedron: Asymmetry 2014 25 489

[109] Liu R.-R Li B.-L Lu J Shen C Gao J.-R Jia Y.-X J. Am. Chem. Soc. 2016 138 5198 27078124 10.1021/jacs.6b01214

[110] Xu R Xia R Luo M Xu X Cheng J Shao X Li Z J. Agric. Food Chem. 2014 62 381 24364696 10.1021/jf4046683

[111] Aron Z D Ito T May T L Overman L E Wang J J. Org. Chem. 2013 78 9929 24090405 10.1021/jo4018099 PMC3843940

[112] Gong X Chen M Yao L Wu J Chem. Asian. J. 2016 11 1613 26991867 10.1002/asia.201600211

[113] Boeckman R K. Jr Genung N E Chen K Ryder T R Org. Lett. 2010 12 1628 20218707 10.1021/ol100397q

[114] Daidone G Sprio V Plescia S Marino M L Bombieri G Bruno G J. Chem. Soc., Perkin Trans. 2 1989 137

[115] Maggio B Raffa D Raimondi M V Cusimano M G Plescia F Cascioferro S Cancemi G Lauricella M Carlisi D Daidone G Eur. J. Med. Chem. 2014 72 1 24333609 10.1016/j.ejmech.2013.11.016

[116] Vicente J Saura-Llamas I Garcıa-López J.-A Bautista D Organometallics 2010 29 4320 10.1021/acs.organomet.0c00787 PMC8895685 35264820

[117] Frutos-Pedreno R Gonzalez-Herrero P Vicente J Organometallics 2012 31 3361

[118] García-Lopez J.-A Oliva-Madrid M.-J Saura-Llamas I Bautista D Vicente J Organometallics 2012 31 6351 10.1021/acs.organomet.0c00787 PMC8895685 35264820

[119] Oliva-Madrid M.-J García-Lopez J.-A Saura-Llamas I Bautista D Vicente J Organometallics 2014 33 6420 10.1021/acs.organomet.0c00787 PMC8895685 35264820

[18a] Borisov R S Voskressensky L G Polyakov A I Borisova T N Varlamov A V Synlett 2014 25 955

[18b] Zubkov F I Ershova J D Orlova A A Zaytsev V P Nikitina E V Peregudov A S Gurbanov A V Borisov R S Khrustalev V N Maharramov A M Varlamov A V Tetrahedron 2009 65 3789

